# A Method for Developing Rapid Screening Values for Active Pharmaceutical Ingredients (APIs) in Water and Results of Initial Application for 119 APIs

**DOI:** 10.3390/ijerph15071308

**Published:** 2018-06-22

**Authors:** Ashley Suchomel, Helen Goeden, Julia Dady

**Affiliations:** Minnesota Department of Health, Saint Paul, MN 55164, USA

**Keywords:** pharmaceuticals, human health, environment, drug labels, screening method, LTD, uncertainty factors, risk assessment, risk context

## Abstract

Americans fill upward of four billion prescriptions for pharmaceuticals each year, and many of those pharmaceuticals eventually make their way into the environment. Hundreds of different active pharmaceutical ingredients (APIs) are detected in ambient waters and source water used for drinking water in the U.S. Very few of these drugs have health-based guidance values that suggest a safe level for individuals exposed in the ambient environment through drinking water. The Minnesota Department of Health (MDH) has developed a novel method to derive screening-level human health guidance values for APIs. This method was designed for rapid evaluation and relies on Food and Drug Administration (FDA)-approved drug labels and limited additional public data resources for necessary information. MDH developed an analytical framework using traditional and novel uncertainty and adjustment factors specific to the information available for APIs. This framework, along with an estimated lowest therapeutic dose (LTD), was used to derive screening reference dose (sRfD) values. Water screening values (WSV) were then derived using the sRfD, a relative source contribution factor (RSC), and a water intake rate for infants to represent a highly exposed population. MDH used this new method to derive water screening values for 119 APIs that are commonly prescribed and/or commonly monitored in Minnesota waters, including antibiotics, antidepressants, steroids, and other classes of drugs. The derived WSVs can be used to provide context to environmental detections, prioritize APIs for further health-based guidance development, prioritize APIs for future environmental monitoring studies, and inform the development or refinement of analytical methods.

## 1. Introduction

In the past twenty-five years, the portion of the United States population that uses at least one prescription pharmaceutical has risen approximately ten percent. From 2011–2014, nearly half of all Americans used at least one prescription medication, and nearly a quarter used three or more [1]. In 2017, upward of four billion prescriptions, or 12.6 prescriptions per capita, were filled by Americans [2]. Trends show that that percentage will continue to grow in the coming years.

The rapid growth in pharmaceutical use has contributed to increased detection of pharmaceuticals in the environment [3,4,5,6]. Many pharmaceuticals are commonly detected in potential drinking water sources and treated drinking water, yet very few of these drugs have established water guidance values that inform the probability of certain health risks associated with large populations consuming water containing prescription drugs [7]. Pharmaceuticals enter the environment through a variety of pathways, including improper disposal down household drains and toilets, disposal into landfills, runoff from manure in agricultural areas after use in animals, industrial releases, and human excretion. While detected environmental concentrations may be lower than other types of contaminants, these compounds are designed to be biologically active and potent at low concentrations, warranting special scrutiny to assess potential human health risk.

Despite the widespread presence of pharmaceuticals in water, these contaminants of emerging concern are not currently regulated for drinking water and wastewater purposes. A number of methodologies have been described in the literature for screening and prioritizing the hazard and risk to human health from pharmaceuticals in the environment [8,9,10,11]. These approaches use various techniques for calculating toxicity values and mainly rely on published data in the literature for derivation of toxicity points of departure and adverse effects. The method developed by the Minnesota Department of Health (MDH) builds on many of these approaches, adding assessment factors that may be of specific concern for pharmaceuticals (e.g., endocrine activity), in addition to incorporating many elements of MDH’s established risk assessment methods to derive water guidance values [12]. A key aspect of the rapid assessment method is a reliance on easily accessible data obtained from the US Food and Drug Administration (FDA) drug labels to provide the majority of the information needed. Pharmaceuticals typically have sufficient safety data as required by FDA; however, much of the data relevant for risk assessment are not available in published literature and drug studies are often considered proprietary. The data source allows for the rapid assessment of a large variety and number of pharmaceuticals that could be present in potential drinking water sources.

The objectives pursued by MDH were: (1) to create a rapid assessment framework for deriving screening reference doses (sRfDs) for orally administered pharmaceuticals using readily available information (e.g., FDA approved drug labels); and (2) to use the sRfDs to derive water screening level values for an initial set of commonly prescribed and detected pharmaceuticals. The developed values can be used for a variety of purposes, including, providing human health context to environmental detections, guiding future monitoring efforts, prioritization of development or refinement of analytical methods, and prioritization the development of health-based standards.

## 2. Methods

### 2.1. Selecting the Most Relevant Pharmaceuticals for Value Development

MDH initially planned to focus on the pharmaceuticals most relevant in Minnesota. However, prescription usage information was not available on a state-specific basis in 2013, when method development was initiated. Information on the top 200 pharmaceuticals most commonly prescribed and used in the United States from 2011–2012 [13,14] was considered representative of pharmaceutical use in Minnesota. This list, which is updated each year, provided an approximation of what may be entering the Minnesota environment, but not necessarily what is actually being found and in what amounts. In addition to the top 200 pharmaceuticals from the national lists, other pharmaceuticals were added if they were included in monitoring efforts relevant to Minnesota and were on common analyte lists from national/federal laboratories. The active pharmaceutical ingredient (API) for each pharmaceutical was identified using information from drug label information found on the National Library of Medicine drug information website DailyMed [15]. Duplicate APIs were removed from the list.

The developed framework provides a method to rapidly derive sRfDs for a large number of APIs based on information from the FDA drug label. The sRfDs are appropriate for most orally administered APIs, with a few exceptions (Table 1). The framework is not designed to address APIs with non-oral routes of administration, genotoxic effects, lack of appropriate FDA labels, and those that are only used for purposes that do not require a prescription for human use. These characteristics were used to establish exclusion criteria. Approximately one-third of the unique APIs were excluded based on the exclusion criteria.

If an appropriate FDA-approved label was found for an API, the description section of the label was searched for possible exclusion criteria. Exclusions based on genotoxic or non-threshold carcinogenicity were generally determined during review of the label and selection of uncertainty and adjustment factors. An unsuccessful search for an applicable FDA-approved label and supporting information usually indicated that one or more of the exclusion criteria applied.

### 2.2. API Data Used for Rapid Assessment

Information for each API was obtained from the most current and appropriate FDA approved label. Labels were accessed via DailyMed [15] and the most recently approved label available for oral administration of the API was selected. Additionally, MDH selected a label for a drug containing the API from an original packager, when available. If a suitable label could not be found for the API in the DailyMed database, the FDA Drugs Database [17] was searched for an applicable label. When information was not available from the drug label, or when additional data was needed to further confirm or support information from the drug label, data was gathered from other sources such as the National Toxicology Program (NTP), FDA New Drug Application (NDA) Data, International Agency for Research on Cancer (IARC), and Hazardous Substances Data Bank (HSDB) [18,19,20].

### 2.3. Lowest Therapeutic Dose Calculation

The lowest therapeutic dose (LTD), the lowest amount of an API that is necessary to produce a clinically effective outcome, was selected as the point of departure (POD) for deriving the sRfDs. The lower end of an API’s therapeutic dosing range can be considered a lower threshold for biological activity, approximating a lowest adverse effect level (LOAEL). The LTD was considered an appropriate POD for the framework because API-related biological effects in the general population were considered undesirable. LTDs based on special dosing for individuals with certain physiological conditions or existing disease (e.g., renal or hepatic impairment), requiring a lower, titrated, or limited dosing, were considered not relevant for the general population and excluded from consideration. Doses on FDA labels were typically expressed as milligram per day (mg/day). In some cases, label instructions indicated dosing was required multiple times per day over various time increments to attain a minimum therapeutic level. In these cases, the LTD included the full amount per an entire 24-h day and not the minimum amount per tablet/capsule (e.g., a 10 mg tablet taken 4 times per day resulted in LTD of 40 mg/day). MDH subsequently calculated the final LTD by converting the selected dose from mg/day to mg/kg-day (Equation (1)):

LTD (mg/kg-day) = Dose of API (mg/kg)/BW based on age (kg)
(1)

An appropriate body weight (BW) in kilograms was selected from the US Environmental Protection Agency (EPA) Exposure Factors Handbook [21,22]. Mean weights by age, as shown in Table 2, that corresponded to the appropriate dosing recommendations from the label were used to calculate the dosage in units of mg/kg-d. If a specific age or body weight range was described on the FDA label (e.g., 12–17 years of age), the LTD calculations were performed on each age group separately (e.g., 12–13, 13–14, etc.) to determine age-group specific LTDs. In these instances, however, the age group with the highest mean weight usually produced the lowest LTD. If doses on the label were already reported in mg/kg-day, no further calculations or adjustments were made.

### 2.4. Uncertainty and Adjustment Factors

Uncertainty factors (UF) and adjustment factors (AF) were used to account for a range of considerations in calculating appropriately conservative sRfDs. While the majority of the UFs applied were based on standard chemical risk assessment methods, modifications to decision criteria for application were developed to better fit specific considerations regarding APIs and the available data from FDA approved labels. Additional AFs were applied to account for special considerations and concerns related to the selected pharmaceuticals, including nonlinear (i.e., threshold) cancer potential and endocrine activity. Unlike MDH’s methodology for developing health-based water guidance following in-depth review, which uses the standard RfD derivation process for nonlinear carcinogens, the MDH rapid assessment methodology for pharmaceuticals addressed carcinogenicity potential with an additional AF for cancer. In total, six potential UF or AFs, represented as UF/AF, may be applied to account for various areas of uncertainty.

**Figure 1:**
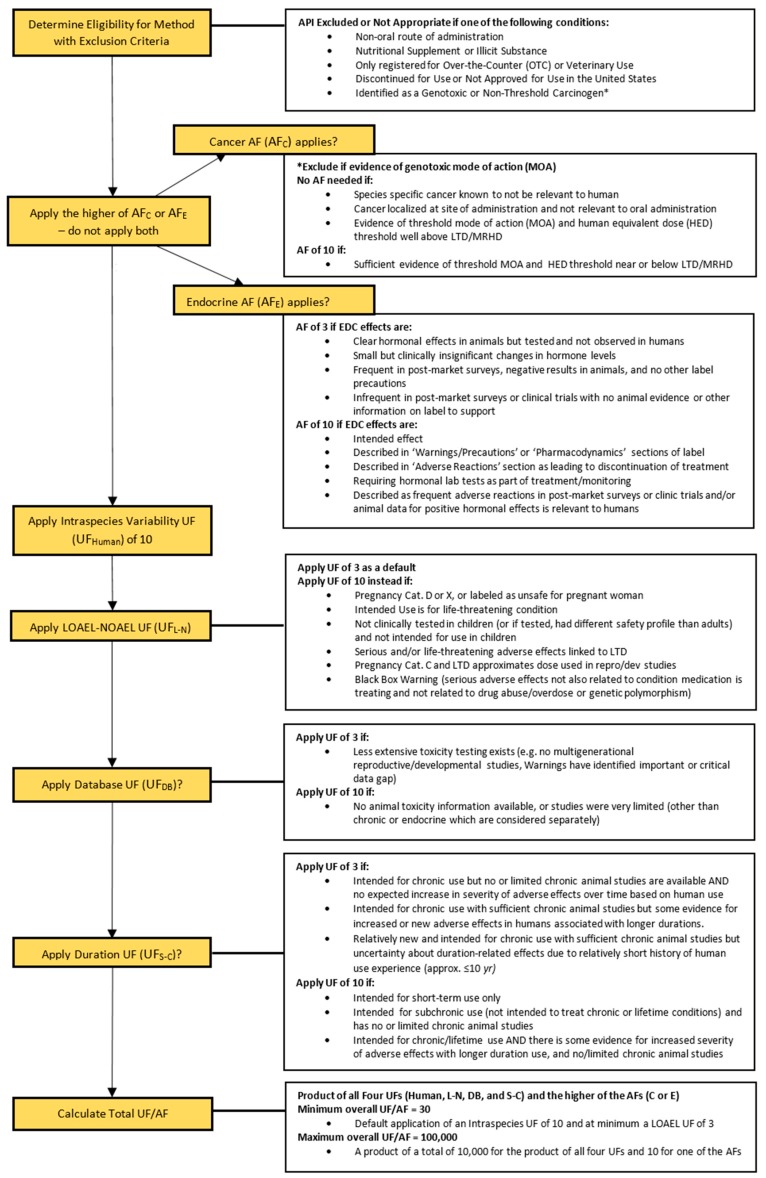
The decision tree provides a summary and guide for the decisions made in regards to application of Adjustment Factors (AFs), Uncertainty Factors (UFs), and calculation of Total UF/AF for use in derivation of a screening reference dose (sRfD).

A Decision Tree was established to facilitate the selection of UFs and AFs (Figure 1). Definitions and guidelines were incorporated into the Decision Tree to ensure consistency in defining and applying the UFs and AFs. Each UF or AF could be assigned a value of 1, 3, or 10. The UF/AF designation was based on the FDA-approved label data for the API or a representative API for the therapeutic class, along with additional sources as needed. A value of 1 was assigned to indicate that the particular UF or AF was not needed for the API. The minimum overall UF/AF possible (product of all six UF/AFs) was 30 (default application of an intraspecies UF of 10 and at minimum a LOAEL UF of 3) and the maximum UF/AF possible was 100,000 (a product of 10,000 for all UFs and 10 for either the AF for endocrine activity or for cancer potential). The rationale for application of specific UFs and AFs is detailed in Section 2.4.1, Section 2.4.2, Section 2.4.3, Section 2.4.4, Section 2.4.5 and Section 2.4.6. Decision criteria for the application of each UF or AF were designed to avoid overlap application of UF/AFs based on the same information. Therefore, application of the maximum total UF/AF of 100,000 was not used in the current assessment and is highly unlikely to occur in future assessments.

Consistent with MDH and EPA risk assessment methodology [12], individual UF/AFs of 3 and 10 were expressed as 30 (3 × 10^1^), whereas individual UF/AFs of 3 and 3 were expressed as 10 (10^0.5^ × 10^0.5^ = 10^1^). For the APIs evaluated, the overall UF/AF was usually at least 100. Each UF and AF is described in more detail in the following subsections and in the Decision Tree (Figure 1).

#### 2.4.1. Cancer Adjustment Factor (AF_C_)

MDH accounted for the risk of potential cancer of an API by applying an AF based on the available information from the FDA label. If the API was determined to be a threshold carcinogen, i.e., a carcinogen for which there is sufficient evidence that a level of exposure exists below which there is no cancer risk, the appropriate Cancer AF (AF_C_) was determined. The FDA labels did not directly state whether or not an API had a threshold or non-threshold mode of action (MOA). Additional literature sources were consulted and professional judgement was required to make determinations. Non-threshold, i.e., linear, carcinogens were not appropriate for this method and were excluded from the evaluation. Application of UFs/AFs to a POD is not appropriate for linear carcinogens. Assessment of linear carcinogens requires access to appropriate cancer study data in order to derive a cancer slope factor rather than deriving an RfD based on a no observable adverse effect level (NOAEL) or LOAEL. For most APIs, appropriately detailed dose-response cancer data were not reported on FDA-approved labels and were not publically available because the studies were considered proprietary. Therefore, application of a rapid assessment method was not possible for linear carcinogens.

For traditional non-linear (threshold) carcinogen risk assessments, MDH evaluates the data to ensure an RfD based on non-cancer effects will also be protective for cancer. A separate cancer-based value using UFs is not typically derived for non-linear carcinogens. In contrast, the rapid screening method was used to derive a sRfD, protective of non-linear cancer effects, by using a cancer adjustment factor (AF_C_). In many cases, drugs tested for carcinogenicity were only found to cause cancer in animals at human equivalent doses (HED) far above the maximum recommended human dose (MRHD). An HED is the human dose estimated to be equivalent to the dose administered to the test animal, based on allometric scaling. For pharmaceuticals, allometric scaling is based on relative body surface area between animals and humans. The carcinogenicity sections on the FDA labels provided the HEDs and comparisons with MRHDs necessary to make a determination about AF_c_. An AF_c_ of 10 was applied when the HED associated with tumors was near or below the MRHD or LTD. If the HED was far above the MRHD or LTD, an AF_c_ was not needed. An AF_c_ was also not applied in cases where the particular type of cancer reported in animals was not relevant to humans (rodent thyroid and liver tumors), or if the cancer was localized at the site of administration and not relevant to oral administration (carcinogenic effects only seen in studies with administration via subcutaneous or intraperitoneal injection and effects not deactivated via first pass metabolism).

#### 2.4.2. Endocrine Activity Adjustment Factor (AF_E_)

MDH accounted for potential adverse effects relating to endocrine activity by applying an Endocrine Activity AF (AF_E_). The AF_E_ was applied when endocrine activity was either the intended effect or a reported side effect of the API. Concerns that the application of the LOAEL-NOAEL UF (UF_L-N_) of 10, discussed in Section 2.4.4, was not adequate to be protective of the very low-level potencies of potential endocrine active APIs warranted the use of this additional AF. MDH considered endocrine activity to include effects related to the female reproductive system, male reproductive system, pituitary gland, adrenal gland, changes in hormones (including estrogen, testosterone, and androgen), and hormonal changes related to the nervous system, blood sugar changes, and metabolism [23]. For endocrine effects aggravated by, but not caused by, the API (e.g., aggravation of diabetes symptoms in diabetic patients), an (AF_E_) was not applied unless an endocrine mode-of-action was identified from the label. Additionally, an AF_E_ was not applied if endocrine effects were described as rare adverse effects on the label, or the API masked signs of endocrine disease by controlling symptoms (e.g., controlled arrhythmias caused by hyperthyroidism). If the AF_C_ was already applied, the AF_E_ was still noted but not additionally applied. This decision was based both in part on the results of other assessment methodologies [10,24] for APIs and to avoid overlapping conservatism in the application of UF/AFs. An Endocrine Activity AF (AF_E_) of 3 was applied when at least one of the following conditions was present on the FDA label or supporting information:

- Clear hormonal effects in animals were observed, but testing in humans was performed and no effects were observed.
- Small but clinically insignificant changes in hormone levels were seen in animal studies.
- Endocrine effects were frequent in post market surveillance in humans but negative endocrine effects were reported in animal studies, and no other precautions for endocrine effects were provided on the label.
- Infrequent endocrine effects in post market surveillance or clinical trials in humans were noted but there were no animal studies available on the label to support the observed endocrine effects.

An Endocrine Activity AF (AF_E_) of 10 was applied when at least one of the following conditions was present on the FDA label or supporting information:

- The endocrine effects observed were the intended therapeutic effects of the API.
- The endocrine effects were described in the ‘Warnings/Precautions’ or ‘Pharmacodynamics’ section of the FDA label.
- The endocrine effects were described in the ‘Adverse Reactions’ section of the FDA label as leading to discontinuation of treatment.
- There were hormonal lab tests that were required or recommended as part of the treatment, or for monitoring individuals taking the API.
- The endocrine effects were described as frequent adverse reactions in post-marketing surveillance or clinical trials and/or there are animal data indicating positive hormonal effects relevant to humans.

#### 2.4.3. Intraspecies Variability Uncertainty Factor (UF_Human_)

MDH accounted for the variation in how human individuals may respond to APIs by applying an Intraspecies Variability UF of 10 to every API. This is consistent with EPA and MDH risk assessment methods for deriving health-based guidance [12,25].

#### 2.4.4. LOAEL-NOAEL (Dosing) Uncertainty Factor (UF_L-N_)

Although APIs are designed to exert a beneficial therapeutic effect on an individual, this effect may be undesirable for the general population. Adverse side effects may also occur at the LTD and drug safety studies may not report or test for effects that occur at doses lower than the LTD. As a result, the LTD was considered to be analogous to a LOAEL and could not be considered as a NOAEL. Therefore, the LOAEL-NOAEL UF (UF_L-N_) was applied.

A LOAEL-NOAEL UF (UF_L-N_) of 3 was applied as a default. The LOAEL-NOAEL UF (UF_L-N_) was increased to 10 if the FDA label or supporting information indicated that the API met any of the following criteria:

- The API was labeled as Pregnancy Category D or X, or labeled as unsafe for pregnant women. FDA pregnancy categories D and X indicate that there could be side effects that may affect sensitive populations at the LTD. Category D is assigned when risks to the fetus were observed in humans, but the benefits may outweigh the risks. Category X indicates studies in animals or humans have shown fetal abnormalities or other risks to the fetus and the risks outweigh the benefits. These two category classifications warrant the use of a more protective UF. In comparison, Categories A and B indicate that adequate information in humans exist that demonstrate no substantial effect to the fetus or that studies failed to demonstrate effects to the fetus and there have been no well-controlled studies in pregnant women. Category C indicates that animal studies have shown adverse effects to the fetus and that there are no adequate studies in humans, but the potential benefits may outweigh the risks.
- The API was labeled as Pregnancy Category C and the LTD approximated the dose used in reproductive or developmental studies that was indicated on the FDA label.
- The API was intended for life threatening conditions. APIs used to treat many serious conditions often have severe side effects that can occur at the level of the LTD. The potential benefits of these APIs may outweigh the risks for those seeking the therapeutic benefit, but the side effects may not be acceptable to the general population.
- The API was not clinically tested in children or, if it was tested in children, it had a different safety profile than adults and the LTD applied only to adults. This extra level of protectiveness was warranted because children are often more sensitive to the effects of APIs.
- The LTD for the API was linked to serious and/or life threatening adverse effects.
- The FDA label for the API contained a black box warning. Certain serious warnings, particularly those that may lead to death or serious injury, are often required to be presented as a black box warning on the label with bold text marked ‘Warning’ [26]. Warnings for which a UF of 10 was applied included serious, life threatening effects not related to the condition or illness that the API was treating. Examples of effects where a UF of 10 was applied include statements concerning drug abuse or overdose, increased suicide from antidepressants, and those related to specific polymorphisms. Potential vulnerability due to genetic polymorphisms is addressed by the Intraspecies UF.

#### 2.4.5. Database Uncertainty Factor (UF_DB_)

A Database UF (UF_DB_) was applied to account for APIs with less extensive toxicity testing, especially if the data gap may be relevant to sensitive populations. A UF_DB_ of 3 was applied to APIs that may have extensive toxicity testing, but an important study appeared to be missing from the drug label. The lack of multigenerational reproductive/developmental studies was a common cause for setting this UF to 3. A UF_DB_ of 10 was applied to APIs that had no animal studies or studies with very limited endpoint testing as described on the available FDA label or other readily available sources (e.g., NTP, HSDB). Additional information may have been available in the published scientific literature, but an extensive literature search and subsequent in-depth analysis and critical review was outside the scope bounds for conducting a rapid assessment because it is very time and resource intensive.

#### 2.4.6. Duration (of Administration) Uncertainty Factor (UF_SC-C_)

A Duration UF (UF_SC-C_) was applied to account for uncertainty based on the length of API use or administration, to account for limited chronic testing, or to account for the potential for increased severity of potential effects over time during the course of taking an API. A Duration UF (UF_SC-C_) of 3 was applied when at least one of the following conditions applied based on the available FDA label or supporting information:

- The API was intended for chronic use (months to years) with no expected increase in severity of adverse effects over time based on extensive time of human use, but had no or limited accessible chronic animal studies.
- The API was intended for chronic use and had sufficient chronic studies in animals, but had some evidence of increased or new risk of adverse effects in humans associated with longer durations of use, including increased risk of dependence on the API.
- The API was intended for chronic use and had sufficient chronic studies in animals, but was relatively new to market, and uncertainty about possible duration-related effects due to a relatively short history of human use remains.

A Duration UF (UF_SC-C_) of 10 was applied when at least one of the following conditions applied based on the available FDA label or supporting information:

- The API was intended for short-term use only (days to weeks)
- The API was intended for subchronic use (months) and had limited or no chronic testing in animals. This includes APIs not intended to treat chronic or lifetime conditions.
- The API was intended for chronic and/or lifetime use with no or limited chronic testing in animals, and there is evidence for increased severity of adverse effects with increasing and longer durations of use.

### 2.5. Screening Reference Dose (sRfD) Calculation

The calculated LTDs, along with the UF/AF assignments, were used in the derivation of sRfDs for each API. The sRfD is calculated in a similar manner to a traditional reference dose (RfD), a daily oral dose that is not likely to have appreciable risk or adverse effects [25,27]. MDH calculated the sRfD by dividing the LTD by the total UF/AF (Equation (2)):

sRfD (mg/kg-d) = LTD (mg/kg-d)/[(AF_C_ or AF_E_) × UF_Human_ × UF_L-N_ × UF_DB_ × UF_S-C_] (2)

### 2.6. Water Screening Value (WSV) Calculation

The WSV was derived using the calculated sRfD, a relative source contribution factor (RSC), a unit conversion factor, and a drinking water intake rate (Equation (3)). The WSV calculation is based on the MDH standard non-cancer assessment algorithm for calculating short-term water guidance values [12]:

WSV (µg/L) = (sRfD (mg/kg-d) × RSC × Conversion Factor (µg/mg))/Water Intake (L/kg-d) WSV (µg/L) = (sRfD (mg/kg-d) × 0.8 × 1000 (µg/mg))/0.289 (L/kg-d)
(3)

Water intake may be only one of several pathways by which an individual may be exposed to a contaminant. An RSC is used to account for exposure other than ingestion of water (e.g., inhalation of volatilized chemicals, dermal absorption) as well as exposure from other media (e.g., diet) to ensure that the cumulative exposure does not exceed the RfD, in this case the sRfD [25]. MDH used the EPA Exposure Decision Tree [25,28] to identify the appropriate RSC value [28]. Within the EPA Decision Tree framework, RSCs can range from 0.2 up to 0.8. The EPA methodology uses a ceiling of 0.8 (80%) and minimum of 0.2 (20%) so that no more than 80% nor less than 20% of the RfD can be accounted for from ingestion of water at the developed guidance value [28]. WSVs were calculated using an RSC of 0.8 for the majority of APIs, based on the assumption that individuals not taking a prescription medication could receive the majority of their exposure through drinking water. An RSC of 0.2 was applied to a very limited number of APIs that have prescription as well as numerous over-the-counter uses, due to concerns regarding the frequency of unintended overdoses [29,30]. An example would be acetaminophen or ibuprofen, which are widely used in infants and children cough, cold and pain medications.

The intake rate used to calculate the WSV is 0.289 L/kg-d, which represents the 95th percentile human infant intake for ages 1–3 months [12]. This is consistent with MDH methodology for completing pesticide rapid assessments and with MDH risk assessment methodology for developing water guidance values [12,31,32]. The use of this intake rate is protective for infants and special susceptible populations, and was considered appropriately conservative for development of screening-level values.

## 3. Results

MDH identified 121 unique APIs from the 200 most prescribed pharmaceuticals in the United States from the 2011 and 2012 Pharmacy Times lists [13,14]. Forty of the 121 unique APIs were excluded from further analysis based on the exclusion criteria outlined in Table 1. Thirty-eight additional unique APIs were identified from monitoring efforts in Minnesota waters. This resulted in a total of 119 APIs for evaluation, 81 from analysis of the top 200 prescribed pharmaceuticals in the United States and 38 additional APIs that were commonly being monitored in Minnesota. Five APIs were included in the assessment even though they did not have current FDA-approved labels or were discontinued for use, despite the general exclusion criteria. These five included: lomefloxacin, norfloxacin, oxytetracycline, propoxyphene, and sulfamethizole. Labels were identified in DailyMed [15] for these five drugs, but it was discovered during the assessment that the API was either recently discontinued for use or the label was too outdated to provide all of the necessary information needed for assessment. In these cases, additional data sources [17,19,20] were relied upon to provide the needed information to derive a sRfD and WSV.

**Figure 2:**
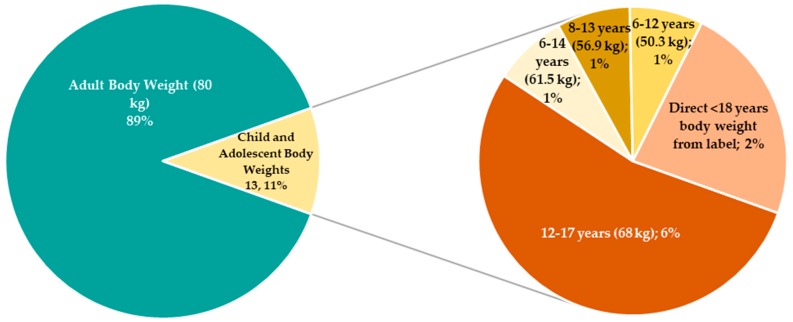
Breakdown of Dosing Basis for Lowest Therapeutic Dose Calculations for the 119 APIs Evaluated. The child and adolescent body weights are based on averages for the ranges [21,22] presented in Table 2.

MDH calculated LTDs for each of the 119 APIs. A large majority (106 or 89%) of calculated LTDs used adult (i.e., ≥18 years of age) dosing, based on recommendations from the label. The remaining 13 (or 11%) used child or adolescent dosing and body weights (BW) from the label to serve as the basis of the LTD (Figure 2). The calculated LTDs ranged from 0.0013 to 25 mg/kg-d, spanning four orders of magnitude.

**Figure 3:**
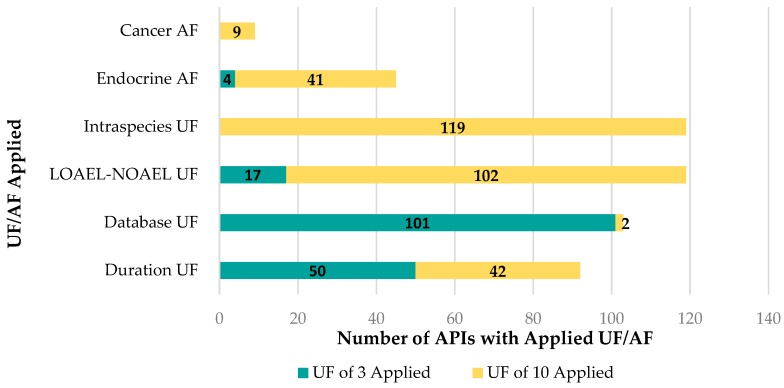
Frequency of Uncertainty (UF) and Adjustment (AF) Factors (described in Section 2.4) Application During Derivation of API sRfD including application of UF/AF of 3 or 10 applied for the Cancer AF (AF_C_), Endocrine AF (AF_E_), Intraspecies UF (UF_Human_), LOAEL-NOAEL UF (UF_L-N_), Database UF (UF_DB_), and the Duration UF (UF_S-C_).

The LTDs were then used to generate sRfDs for 119 APIs. The total UF/AF adjustment applied to the 119 APIs ranged from 100 to 30,000. The Intraspecies UF (UF_Human_) of 10 was applied to all APIs. The LOAEL-NOAEL UF (UF_L-N_) of 3 or 10 was applied to all APIs as well. A breakdown of the frequency of application for each UF and AF is shown in Figure 3.

A UF_L-N_ of 10 was applied to the majority of APIs (102 out of 119 or 86%) and a UF_L-N_ of 3 was applied to the remaining 17 APIs (14%) as a default UF. Often, multiple decision points for application of a UF_L-N_ of 10 applied to each API, such as a black box warning being present on the label and the API not being intended for use in children. These instances were recorded, but only one UF_L-N_ was applied in these instances. Relevant black box warnings or pregnancy category D or X statements were found on labels for 42% of APIs meeting the criteria for application of a UF_L-N._ The label for 24% of APIs specifically indicated that the API was not intended for use in children, meeting the criteria for application of a UF_L-N_. Serious adverse effects occurring at the LTD were noted on the label for 15% of APIs and 14% were labeled with a Pregnancy Category C classification with an LTD that approximated doses used in reproductive studies, both cases also meeting the criteria for application of a UF_L-N_ of 10.

The UF_S-C_ was applied to 92 of 119 (77%) of APIs. The UF_S-C_ of 3 was applied slightly more often (50 out of 92 or 54%) than the UF_S-C_ of 10 (42 out of 92 or 46%). The UF_S-C_ of 3 was typically applied to account for intended chronic use of an API that had sufficient testing apparent on the label or supporting information, but there was evidence of an increase in incidence or severity of effects with increased duration of use. The UF_S-C_ of 10 was most often applied to account for APIs intended for short-term use (e.g., antibiotics, pain relievers, and sedatives).

A UF_DB_ of 3 or 10 was applied to 103 of 119 (87%) of APIs. The UF_DB of_ 3 was most often applied (101 out of 103 or 98%) to account for a lack of multigenerational study to appropriately qualify reproductive and developmental risks. The UF_DB of_ 10 was applied to two APIs (2%), benztropine and digoxin, to account for a lack of animal studies, most notably for reproductive and developmental endpoints, being described on the label or supporting information. 

The framework for deriving sRfDs included two novel adjustment factors: Cancer AF (AF_C_) and Endocrine (AF_E_). These adjustment factors were applied to less than half of the 119 APIs. In cases where both factors were deemed appropriate only the higher of the two AFs was applied in deriving the sRfD. The AF_C_ of 10 was applied to nine APIs (digoxin, drospirenone, fenofibrate, gemofibrozil, olanzapine, pioglitazone, primidone, quetiapine, and risperidone) to account for evidence of a threshold carcinogenic mode of action with the threshold near or below the LTD or MRHD. The cancer endpoints identified for these nine APIs included liver carcinomas and tumors, mammary gland and interstitial testes tumors, bladder tumors, thyroid cancers, and adrenal gland tumors. 

The AF_E_ was applied to 45 APIs, with the application of a AF_E_ of 10 being the most frequently applied value (41 out of 45). The AF_E_ of 10 was usually applied for one of two reasons: (1) for 61% (25 out of 41) it was based on endocrine side-effects (e.g., antidiuretic hormone effects, increased hormone levels, goitrogenic effects, gynecomastia) described in the warnings and precautions, pharmacodynamics, and adverse reactions sections of the label; or (2) for the remaining 39% (16 of 41) it was based on endocrine effects that were the intended therapeutic effect (e.g., insulin stimulation, regulation of thyroid activity) for APIs that were glucocorticoids, antidepressants, hormones, or lipid lowering drugs. The AF_E_ of 3, on the other hand, was usually applied to account for infrequent endocrine effects (hormone level changes) being reported in humans with no animal data to support these observed effects.

Some individual APIs lacked data to adequately evaluate carcinogenic potential or endocrine activity. During the assessment it was determined that APIs within the same class could be used to address these data gaps. Three classes of APIs (i.e., statins, sulfonamides, and tetracyclines) were assessed as a group for determining the appropriate application of the Cancer AF and/or Endocrine Activity AF. A group assessment was considered appropriate since the general modes-of-action are similar within each class.

Statins, including atorvastatin, lovastatin, pravastatin, rosuvastatin, and simvastatin, were each assigned an AF_E_ of 10 based on clear indications for endocrine effects on the ‘Warnings and Precautions’ section of the label. Sulfonamides, including sulfadiazine, sulfamethizole, and sulfamethoxazole, were each assigned an AF_E_ of 10 to account for known drug class effects on the thyroid. The tetracycline drug class, including demeclocycline, doxycycline, minocycline, oxytetracycline, and tetracycline, were reviewed together to determine Cancer and Endocrine AFs. The tetracycline class evaluation was based on the label for minocycline and supporting information on labels for others in the group. Tetracyclines were assigned an AF_E_ of 10 for thyroid effects including thyroid hyperplasia and the potential of thyroid related cancers. An AF_C_ of 1 was assigned to the five tetracyclines based on the lack of information provided on labels to determine relative dosing related to human doses for cancer studies, and due to the likely cancer mechanism, if present, being related to endocrine mechanisms, which was more appropriately covered with the application of the AF_E_.

The sRfDs were calculated using the LTD and overall UF (Equation (2)). Each sRfD along with an RSC and an infant water intake rate were used to generate the WSV (Equation (3)). An RSC of 0.8 was used for the majority of the 119 APIs. However, an RSC of 0.2 was used for one API, ibuprofen, because it is included in multiple over-the-counter (OTC) formulations intended for children in addition to prescription pharmaceuticals. The derived sRfDs ranged from 0.00000016 mg/kg-d to 0.12 mg/kg-d, spanning six orders of magnitude. The final derived water screening values (Table 3) also spanned six orders of magnitude with values ranging from 0.0004 µg/L to 400 µg/L. A detailed breakdown of the calculation for each of the 119 APIs (i.e., LTD, sRfD, WSV, UF/AF application, and reference label sources) is provided in Table S1. Pharmaceutical Water Screening Values Table (see supplementary information).

## 4. Discussion

MDH developed a novel method to rapidly derive screening level values for APIs that have the potential to be found in the environment. As described in Section 2, this method relied upon established MDH risk assessment practices as well as pharmaceutical specific data to derive appropriately conservative water screening values in a rapid manner.

### 4.1. Data Sources 

The selection of an appropriate FDA label was key to deriving a WSV for each API. As previously mentioned, it was important to find the most current and active FDA label available for oral administration of the API. Labels from the original packager and brand names, that were no more than three years old, were used to derive the screening values in 2014. When original labels were not available, repackagers and generic labels were used. When labels in DailyMed [15] were older than two years, the FDA Drugs Database [17] was consulted to confirm that it was the most up-to-date available label.

The available FDA-approved labels can change over time due to new FDA labeling requirements or availability of new safety data. Changes to labeling requirements could affect where relevant information is found on the label and could change how the available data is interpreted. During development of the 119 screening values, the FDA finalized a new Pregnancy and Lactation Labeling Rule (PLLR) that changed how pregnancy and lactation data were presented on the label [33]. The new rule removed the use of pregnancy letter categories (A, B, C, D, and X) and replaced it with three subsections labeled ‘Pregnancy’, ‘Lactation’, and ‘Females and Males of Reproductive Potential’. This new rule gradually phased in the new requirements for existing products and immediately impacted newly registered products. These changes do not impact the values already derived; however, the evaluation of new products will have to be slightly altered to fit the new label presentation. The developed methodology is still useful when evaluating these new products, as the description of effects will still be available. However, the pregnancy letter categories which made the LOAEL-NOAEL UF designation easy to apply, will not be present.

Additional changes to other label sections may occur if additional rule changes are made by the FDA. Changes to labels may also occur as new safety data are added. When there is updated safety information, the FDA [34] generally directs that relevant labels be updated to reflect those changes. MDH, however, found that labels in DailyMed [15] were not always updated. Changes to labels can also be due to changes in manufacturer or packager of the API, as well as formulations being acquired by different companies as a product becomes generic. DailyMed [15] did not have an archive process for these labels when a new label was issued under the same manufacturer at the time of the MDH assessment. For these reasons, labels for assessment were chosen carefully and were identified during value derivation (Table S1). With this knowledge, MDH relied heavily on the FDA-approved label, but other sources were consulted to verify information and fill in data gaps.

The data sources used for this rapid assessment method placed constraints on the types of APIs that could be evaluated. Over-the-counter drugs (OTC), genotoxic or non-threshold carcinogens, and those with non-oral routes of administration cannot be adequately assessed using this method, even though they are often detected in the environment. The Decision Tree in Figure 1 could be easily adapted for OTC drugs; however, different sources of relevant information would need to be identified to facilitate consistent and rapid reviews since OTC labels do not contain the same level of detail as prescription labels. Appropriate risk assessments of genotoxic and non-linear carcinogens require development of cancer slope factors. However, cancer slope factors are not available on FDA-approved labels and cannot be developed without access to data that are currently not publicly available. Drugs with non-oral routes of administration need to be assessed using route-to-route extrapolation. While there are various methods available to conduct route-to-route extrapolation, there are currently no methods developed to facilitate a rapid screening assessment for non-orally administered APIs. Additional or different methods, decision criteria data, and data sources would need be needed to address these groups of APIs.

### 4.2. Appropriately Conservative Methodology

The rapid assessment method was designed to derive appropriately protective sRfDs and WSVs. The method is designed to be more conservative than values generated using the established MDH methodology for in-depth chemical reviews. The level of protectiveness is appropriate for a screening value meant to protect the general population, including sensitive or highly exposed populations, based on limited data or time for assessment. To ensure that the sRfDs and WSVs were sufficiently protective, appropriately conservative selections were made for a variety of parameters used to derive sRfDs and WSVs.

An adult body weight of 80 kg was used in the LTD calculations. As seen in Figure 2, the most common body weight used in the development of the LTDs was the 80 kg adult body weight. In most risk assessment methodologies, 70 kg is the standard adult body weight. According to the EPA, the average adult body weight has increased in recent years, making the 80 kg estimate more appropriate for the current general US adult population [22]. Use of the higher adult body weight results in lower LTDs and sRfD.

The Decision Tree (Figure 1) for application of UF/AFs based on drug label and supporting information was designed to ensure that the resulting sRfD, which was based on limited data and level of evaluation, would be protective of the most sensitive members of the general population. MDH risk assessment methodology for conducting in-depth chemical reviews, uses a maximum UF of 3000 when deriving an RfDs [12]. A chemical with a UF over 3000 is deemed to have insufficient information to derive an appropriate health-based value. The rapid assessment methodology resulted in the application of overall UFs ranging from 100 to 30,000, with 33 (28%) having values greater than 3000. The majority of APIs with a total UF greater than 3000 were either endocrine-active or had a LOAEL-NOAEL UF (UF_L-N_) of 10 (Table S1). The common application of an UF_L-N_ of 10 is not unexpected given that APIs are designed to be biologically active at low doses.

Uncertainty factors for intraspecies variability (UF_HUMAN_), LOAEL-NOAEL extrapolation (UF_L-N_), database deficiencies (UF_DB_), and duration (UF_S-to-C_) are commonly applied in established risk assessment practices for industrial and commercial product environmental contaminants. Unlike APIs, these environmental contaminants are not usually designed to be biologically active in humans. To account for this intended biological activity, MDH included additional adjustment factors for cancer (AF_C_) and endocrine activity potential (AF_E_) in the rapid assessment framework for pharmaceuticals. The use of additional factors for cancer and endocrine activity has precedence based on other methods and approaches described in the published literature [10,24]. The Australian government (AU) has published guidelines for water recycling that include development of surrogate acceptable daily intakes for pharmaceuticals [24]. The AU describes use of a 10-fold safety factor for hormonally active steroids because normal hormone function and fertility could be adversely affected in those not taking the medication for therapeutic benefits [24]. This hormonally-based safety factor used by the AU is similar to the endocrine activity AF applied in MDH’s rapid assessment methodology. The WateReuse Foundation also identified hormonally active compounds and genotoxic carcinogens to be of particular concern and incorporated a UF of 10 in their assessment [10]. These additional AFs provided an extra degree of protection for effects that were not necessarily captured by the other UFs, and may be related to the intended biological effect of the API. The maximum LOAEL-NOAEL UF of 10 may not be adequately protective for APIs designed to effect endocrine targets or for potential carcinogens.

The use of a water intake rate based on bottle-fed infants of 0.289 L/kg-d also added to the conservative nature of the derived screening values. Bottle-fed infants have a higher intake of water on a per body weight basis than individuals at any other life-stage, and are more likely to ingest a higher dose than adults [12,32]. The high infant intake rate is protective of formula-fed infants as well as other sensitive populations. The same intake rate and rationale has been applied by MDH for deriving rapid assessment values for pesticides [31] and is recommended for screening level values [32].

#### Comparison of WSVs with MDH Derived Health-Based Guidance (HBG)

To test the developed method, MDH compared the WSVs derived using the rapid assessment methodology with health-based guidance values (HBGs) derived from traditional in-depth reviews for five APIs [35]). The five APIs were acetaminophen [36], carbamazepine [37], 17a-ethinylestradiol [38], sulfamethoxazole [39], and venlafaxine [40]. The HBGs are based on an in-depth evaluation of potential health risk and are preferred over WSVs when available.

The WSVs derived using the rapid assessment methodology were 2 to 250 times lower, or more conservative, than HBGs derived using established MDH in-depth review methods (Table 4). For four of the five (acetaminophen, carbamazepine, sulfamethoxazole, and venlafaxine) APIs the same RSC (0.2 for acetaminophen and 0.8 for the others) and intake rate (0.289 L/kg-d) were used to derive both the WSV and the HBG. When these inputs were the same, the resulting WSVs were lower than HBGs due, in part, to use of the LTD as the point of departure (POD) instead of a LOAEL or NOAEL, as well as of the additional UFs and AFs. Total UF/AFs for WSVs were higher than those used for HBGs. This was expected because full in-depth reviews required for HBG development were more refined and involved critical examination of much larger datasets, which reduced the degree of uncertainty.

For the remaining API, 17a-ethinylestradiol, the RSC used to derive the WSV and HBG was 0.8, but the intake rates differed. The HBG derived from a full in-depth review supported use of a lower, sub-chronic water intake rate (0.070 L/kg-d) rather than the infant intake rate (0.289 L/kg-d) used in deriving the WSV. The lower intake rate in the HBG calculation and the higher overall UF applied in the WSV calculation resulted in a nearly identical values. This indicated that even for hormonal active compounds that mimic endogenous chemicals, the derived WSVs are near or lower than values derived with traditional methods.

The two WSVs for acetaminophen represented the recommended daily dose ranges for different therapeutic purposes. For example, a simple headache might be treated effectively with only one tablet but more severe pain or chronic conditions such as arthritis might require the maximum recommended daily dosing of six tablets (i.e., 1 tablet every 4 h). The lower LTD of 3.75 mg/kg-d and WSV of 9 µg/L were based on one tablet per day while the higher LTD of 22.5 mg/kg-d and WSV of 50 µg/L were based on six tablets per day.

The limited comparison of rapid assessment-based WSVs and HBGs, which are based on an in-depth review, demonstrates a reasonable level of conservatism. Therefore, WSVs were considered appropriate for screening and prioritization purposes and were not likely to underestimate risk.

### 4.3. Applications of the Values and Use of the Method

The WSV derived by MDH using the rapid assessment methodology are most appropriate for prioritization and screening purposes. MDH recommends using WSVs as a first tier assessment for detections of APIs in a variety of water environments, including surface water, groundwater, and treated drinking water. Ingestion of water is unlikely to pose a threat to human health when API water concentrations are below the WSV. WSVs can also be used for: (1) setting priorities for deriving new health-based guidance based on in-depth reviews; (2) setting priorities for developing new or improved laboratory analytical methods; (3) selecting APIs to be included in future monitoring projects; and (4) assisting in the evaluation of water quality. Situations in which water detections exceed the WSV, may benefit from completing a more thorough risk assessment for the API. Many APIs may not have any available analytical methods, making it impossible or difficult for them to be included in environmental monitoring programs. The WSVs may help to identify APIs that warrant development of new or improved analytical methods. The water screening values are not designed to be used as definitive estimates of risk. A more refined assessment, including detailed toxicity and exposure evaluations, should be done before specific risk management decisions are made.

To date, MDH has used the derived values to provide context to environmental detections of various monitoring studies of surface water and groundwater in Minnesota. The majority of detections have been below developed WSVs. Only two APIs, hydrochlorothiazide (WSV of 0.04 ug/L) and methylprednisolone (WSV of 0.005 ug/L), have been detected in Minnesota surface water at concentrations exceeding the WSV (0.0571 ug/L and 0.006, respectively) [41,42]. No concentrations for APIs in Minnesota groundwater have exceeded a WSV. Gabapentin, an anticonvulsant is detected frequently and at relatively high concentrations compared to other APIs in Minnesota surface waters, sometimes at concentrations over 1 ug/L [42]. While these concentrations appear of concern in contrast to concentrations of other APIs, the concentrations are well below the developed WSV of 300 ug/L indicating that gabapentin detections are not likely to pose a human health concern at detected concentrations. It should be noted that WSVs may not be protective of ecological receptors.

Although, concentrations did not exceed the WSV for most APIs in Minnesota, that does not indicate that concentrations of APIs in other areas and states are not of potential concern. The WSVs can be used to provide context to environmental detections in waters throughout the country to similarly prioritize monitoring efforts and determine potential health risk posed by detected concentrations.

Additionally, MDH compared the WSVs to available maximum detection limits (MDL) and maximum reporting limits (MRL) for analytical schedules from EPA (Methods 1694, 1698) [43,44], USGS (Methods 2434, 2440, and 2080) [45,46,47], and SGS Axys Analytical pharmaceutical methods [48], all of which are commonly used to analyze Minnesota monitoring samples. The MDL or MRL exceeded the WSV for eight APIs, including benztropine, digoxin, glyburide, hydrocortisone, methylprednisolone, oxycodone, prednisolone, and prednisone, indicating that efforts to improve (lower) detection limits of existing analytical methods may be warranted.

MDH was also unable to find evidence of monitoring capability for nearly 22% (18 of 81) of the most commonly prescribed APIs. Given the potential for environmental release development of analytical capabilities should be a research priority.

## 5. Conclusions

MDH has developed a rapid assessment method for deriving WSVs for APIs. This approach is rooted in traditional risk assessment practices and builds upon related methods created by other organizations. This method can be applied for most orally administered human prescription drugs. Screening level values were developed relatively quickly using data from FDA-approved drug labels. MDH used the rapid assessment method to derive sRfDs and WSVs for 119 unique APIs that are commonly prescribed and/or monitored in the environment. The use of FDA-approved labels and limited additional sources allowed for the derivation of consistent and appropriately conservative screening level values. These screening values filled existing data gaps in the available guidance for many of these APIs.

Over four billion pharmaceutical prescriptions were filled last year in the United States, and this estimate is expected to increase in coming years. Continually increasing trends in prescription usage means that APIs have an ever-growing presence in the natural environment. Pharmaceuticals are nearly ubiquitous in most environmental media as a result of improper disposal and normal human excretion. Growing concerns about widespread prevalence of APIs in the environment led to the realization that a rapid method for developing values to provide context for the occurrence of APIs in the environment was required. The rapid assessment method and screening values developed by MDH provide information that can be used to respond to current detections and allow risk managers opportunities to be proactive in setting future priorities. Some future priorities include continued monitoring for pharmaceuticals in environmental media, setting priorities for more detailed and impactful pharmaceutical risk assessments, and identifying the need for new or modified analytical methods.

## Figures and Tables

**Table 1:** Exclusion Criteria for Applicable APIs.

Exclusion Criteria	Description
Non-oral route of administration	The bioavailability of an API given orally differs from that of an API given via another route of administration. Route to route modifications would be necessary to adjust for a non-oral route. The MDH method was designed to rapidly derive water screening values (WSV) related to oral ingestion, only. APIs designed to be administered vaginally, dermally, sublingually, via suppository, via injection (intraperitoneally, subcutaneously, or intravenously), and via inhalation are not appropriate for this method.
Nutritional supplement	The acceptable daily intake (ADI) values and dietary reference intake (DRI) levels for nutrients found in food and pharmaceuticals are available and more appropriate for to deriving human-health based guidance values than the calculated lowest therapeutic doses (LTD) used in this method [16].
Over-the-Counter (OTC) medication only	Labels for OTC drugs do not provide the necessary information to use the developed method.
Illicit Substance	Most illicit substances do not have an FDA-approved label. Some illicit substances may be used for therapeutic purposes with a prescription; however, the potential adverse effects may not be appropriate for analysis with this method.
Discontinued or Not Approved in US	Many discontinued products no longer have active FDA-approved labels that contain the necessary information to use the developed method. If the drug is not approved for use in the US, then it is not likely to be found in US waters in significant quantities.
Registered for Veterinary Purposes only	Labels for veterinary use APIs are not always required to provide the same level of detail as labels with APIs intended for human use.
Genotoxic or Non-Threshold Carcinogen	The developed method may not be adequate to derive an appropriately conservative screening reference doses (sRfD) or WSVs for genotoxic or non-threshold carcinogens.

**Table 2:** Mean Weights by Age Used in Lowest Therapeutic Dose Calculations.

Age (Years)	Mean Weight (kg) ^1^
6–7 ^1^	22.5
7–8	27.4
8–9	31.3
9–10	36.2
10–11	39.5
11–12	44.6
12–13	50.3
13–14	56.9
14–15	61.5
15–16	65.9
16–17	68.0
17–18	66.6
≥18 ^2^	80

Selected mean weights were based on the dosing age range presented on the drug label. ^1^ Dosing for ages under 6 years of age normally reported as mg/kg with no need for further calculation. ^2^ The mean weight (kg) for adults 18+ years of age is from EPA Exposure Factors Handbook 2011 Edition Table 8-1, comprising 1999–2006 data [22]. Increased weight reflects the higher average weight of a United States adult and adds to conservativeness of calculations.

**Table 3:** Derived Water Screening Values (WSV), Screening Reference Doses (sRfD), Uncertainty and Adjustment Factor (UF/AF) application, and calculated Lowest Therapeutic Doses (LTD) for 119 APIs ^1^.

API	CASRN	LTD (mg/kg)	Total UF/AF	sRfD (mg/kg-d)	WSV (µg/L)
Albuterol	18559-94-9	0.075	100	0.00075	2
Alendronate	66376-36-1	0.063	300	0.00021	0.6
Allopurinol	315-30-0	2.50	100	0.025	70
Alprazolam	28981-97-7	0.0094	1000	0.0000094	0.03
Amitriptyline	50-48-6	0.74	10000	0.000074	0.2
Amlodipine	88150-42-9	0.037	100	0.00037	1
Amoxicillin	26787-78-0	12.5	1000	0.013	40
Amphetamine salts	-	0.039	3000	0.000013	0.04
Ampicillin	69-53-4	12.5	3000	0.0042	10
Atenolol	29122-68-7	0.63	1000	0.00063	2
Atorvastatin	134523-00-5	0.13	3000	0.000043	0.1
Azithromycin	83905-01-5	3.13	3000	0.001	3
Benztropine	86-13-5	0.013	3000	0.0000043	0.01
Betaxolol	63659-18-7	0.13	1000	0.00013	0.4
Bisoprolol	66722-44-9	0.6	1000	0.0006	2
Carisoprodol	78-44-4	9.38	1000	0.0094	30
Carvedilol	72956-09-3	0.313	300	0.001	3
Celecoxib	169590-42-5	2.50	1000	0.0025	7
Cephalexin	15686-71-2	12.5	1000	0.013	40
Cimetidine	51481-61-9	10	1000	0.01	30
Ciprofloxacin	85721-33-1	6.25	3000	0.0021	6
Clarithromycin	81103-11-9	6.25	3000	0.0021	6
Clavulanate	58001-44-8	3.13	1000	0.0031	9
Clindamycin	18323-44-9	7.50	3000	0.0025	7
Clonazepam	1622-61-3	0.013	3000	0.0000043	0.01
Clonidine	4205-90-7	0.0025	300	0.0000083	0.02
Clopidogrel	113665-84-2	0.94	300	0.0031	9
Codeine	76-57-3	0.19	1000	0.00019	0.5
Cyclobenzaprine	303-53-7	0.19	10000	0.000019	0.05
Demeclocycline	127-33-3	7	30000	0.00023	0.6
Diazepam	439-14-5	0.044	300	0.00015	0.4
Diclofenac	15307-86-5	1.25	300	0.0042	10
Digoxin	20830-75-5	0.0016	10000	0.00000016	0.0004
Diltiazem	42399-41-7	1.5	1000	0.0015	4
Doxepin	1668-19-5	0.94	3000	0.00032	0.9
Doxycycline	564-25-0	0.91	30000	0.000030	0.08
Drospirenone	67392-87-4	0.038	10000	0.0000038	0.01
Duloxetine	116539-59-4	0.50	1000	0.0005	1
Enalapril	75847-73-3	0.063	300	0.00021	0.6
Erythromycin	114-07-8	12.5	1000	0.013	40
Escitalopram	128196-01-0	0.13	3000	0.000043	0.1
Ezetimibe	163222-33-1	0.125	300	0.00042	1
Fenofibrate	49562-28-9	0.60	3000	0.0002	0.6
Fenoprofen	31879-05-7	2.5	3000	0.00083	2
Fluconazole	86386-73-4	1.25	10000	0.00013	0.4
Fluoxetine	54910-89-3	0.25	3000	0.000083	0.2
Furosemide	54-31-9	0.25	300	0.00083	2
Gabapentin	60142-96-3	11.3	100	0.11	300
Gemfibrozil	25812-30-0	15	3000	0.005	10
Glipizide	29094-61-9	0.19	10000	0.000019	0.05
Glyburide	10238-21-8	0.016	10000	0.0000016	0.004
Hydrochlorothiazide	58-93-5	0.16	10000	0.000016	0.04
Hydrocodone	125-29-1	0.25	10000	0.000025	0.07
Hydrocortisone	50-23-7	0.25	30000	0.0000083	0.02
Ibuprofen	15687-27-1	20	3000	0.0067	5^i^
Imipramine	50-49-7	0.37	10000	0.000037	0.1
Indomethacin	53-86-1	0.63	300	0.0021	6
Ketoprofen	22071-15-4	0.94	1000	0.00094	3
Lamotrigine	84057-84-1	2.81	300	0.0094	30
Levothyroxine	51-48-9	0.0013	300	0.0000043	0.01
Lisdexamfetamine	608137-32-2	0.38	3000	0.00013	0.4
Lisinopril	76547-98-3	0.063	3000	0.000021	0.06
Lomefloxacin	98079-51-7	20	3000	0.0067	20
Lorazepam	846-49-1	0.025	3000	0.0000083	0.02
Losartan	114798-26-4	0.63	300	0.0021	6
Lovastatin	75330-75-5	0.13	10000	0.000013	0.04
Mefenamic acid	61-68-7	12.5	3000	0.0042	10
Meloxicam	71125-38-7	0.094	1000	0.000094	0.3
Memantine	19982-08-2	0.25	1000	0.00025	0.7
Meprobamate	57-53-4	3.98	1000	0.004	10
Metformin	657-24-9	14.7	10000	0.0015	4
Methylphenidate	113-45-1	0.25	1000	0.00025	0.7
Methylprednisolone	83-43-2	0.05	30000	0.0000017	0.005
Metoprolol	51384-51-1	0.31	300	0.001	3
Minocycline	10118-90-8	2.5	30000	0.000083	0.2
Montelukast	158966-92-8	0.081	100	0.00081	2
Naproxen	22204-53-1	6.25	1000	0.0063	20
Nebivolol	118457-14-0	0.031	1000	0.000031	0.09
Nifedipine	21829-25-4	0.38	1000	0.00038	1
Norfloxacin	70458-96-7	10	3000	0.0033	10
Ofloxacin	82419-36-1	5	3000	0.0017	5
Olanzapine	132539-06-1	0.037	10000	0.0000037	0.01
Olmesartan medoxomil	144689-63-4	0.25	300	0.00083	2
Oxycodone	76-42-6	0.25	30000	0.0000083	0.02
Oxytetracycline	79-57-2	6.25	30000	0.00021	0.6
Penicillin V	87-08-1	9.38	3000	0.0031	9
Pentoxyifylline	6493-05-6	5	300	0.017	50
Pioglitazone	111025-46-8	0.19	10000	0.000019	0.05
Pravastatin	81093-37-0	0.35	10000	0.000035	0.1
Prednisolone	50-24-8	0.06	30000	0.000002	0.006
Prednisone	53-03-2	0.063	30000	0.0000021	0.006
Pregabalin	148553-50-8	1.88	300	0.0063	20
Primidone	125-33-7	9.38	3000	0.0031	9
Progesterone	57-83-0	2.5	30000	0.000083	0.2
Promethazine	60-87-7	0.23	3000	0.000077	0.2
Propranolol	525-66-6	0.38	3000	0.00013	0.4
Propoxyphene	469-62-5	4.88	3000	0.0016	4
Quetiapine	111974-69-7	0.63	10000	0.000063	0.2
Ranitidine	66357-35-5	2	100	0.02	60
Risperidone	106266-06-2	0.0074	3000	0.0000025	0.007
Rosuvastatin	287714-41-4	0.063	10000	0.0000063	0.02
Sertraline	79617-96-2	0.31	3000	0.0001	0.3
Sildenafil	139755-83-2	0.13	1000	0.00013	0.4
Simvastatin	79902-63-9	0.063	10000	0.0000063	0.02
Sitagliptin	486460-32-6	1.25	10000	0.00013	0.4
Sulfadiazine	68-35-9	25	10000	0.0025	7
Sulfamethizole	144-82-1	12.5	30000	0.00042	1
Tadalafil	171596-29-5	0.031	300	0.0001	0.3
Tamsulosin	106133-20-4	0.005	3000	0.0000017	0.005
Temazepam	846-50-4	0.09	3000	0.00003	0.08
Tetracycline	60-54-8	18.8	30000	0.00063	2
Tramadol	27203-92-5	2.50	1000	0.0025	7
Trazodone	19794-93-5	1.88	10000	0.00019	0.5
Triamterene	396-01-0	0.47	300	0.0016	4
Trimethoprim	738-70-5	4.57	3000	0.0015	4
Valsartan	137862-53-4	1	300	0.0033	9
Verapamil	52-53-9	2.25	1000	0.0023	6
Warfarin	81-81-2	0.025	1000	0.000025	0.07
Zolpidem	82626-48-0	0.063	3000	0.000021	0.06

^1^ Derived values are based on data and drug labels accessed in 2014 from DailyMed [15] and the FDA Drug Database [17].

**Table 4:** Comparison of Inputs and Values for the Pharmaceutical Rapid Assessment Method for Deriving WSVs and MDH established traditional method for deriving Health-Based Guidance Values (HBG).

API	WSV (µg/L)	MDH HBG (µg/L)	Level of Protection	Pharmaceutical Rapid Assessment Method Inputs	MDH Guidance Value Inputs ^1^
Acetaminophen ^2^	9 50	200	4-22x	LTD1—3.75 LTD2—22.5 mg/kg-d UF_HUMAN_—10 UF_L-N_—10 UF_DB_—3 UF_S-C_—10 Overall UF/AF—3000	POD—7.4 mg/kg-d UF_H_—10 UF_DB_—3 Total UF—300
Carbamazepine	0.9	40	44x	LTD—1 mg/kg-d AF_C_—10 UF_HUMAN_—10 UF_L-N_—10 UF_S-C_—3 Overall UF/AF—3000	POD—3.8 mg/kg-d UF_H_—10 UF_L-N_—10 UF_DB_—3 Total UF—300
17a-Ethinylestradiol	0.0001	0.0002	2x	LTD—0.00044 mg/kg-d AF_E_—10 UF_HUMAN_—10 UF_L-N_—10 UF_DB_—3 UF_S-C_—3 Overall UF/AF—3000	POD—4.2 × 10^−7^ mg/kg-d UF_H_—10 UF_A_—3 Total UF—30
Sulfamethoxazole	0.4	100	250x	LTD—4.57 mg/kg-d AF_E_—10 UF_HUMAN_—10 UF_L-N_—10 UF_DB_—3 UF_S-C_—10 Overall UF/AF—30,000	POD—1.2 mg/kg-d UF_H_—10 UF_A_—3 Overall UF—30
Venlafaxine	0.3	10	33x	LTD—25 mg/kg-d UF_HUMAN_—10 UF_L-N_—10 UF_DB_—3 UF_S-C_—10 Overall UF/AF—3000	POD—0.54 mg/kg-d UF_H_—10 UF_L-N_—10 Total UF—100

^1^ Points-of-departure (POD) for derving MDH Health-Based Guidance (HBG) are NOAELS or LOAELS. Uncertainty factors (UF) used in deriving HBGs include Intraspecies UF (UF_H_), Interspecies UF (UF_A_), LOAEL-NOAEL UF (UF_L-N_), and a Database UF (UF_DB_). ^2^ The lower LTD of 3.75 mg/kg-d (LTD_1_) and WSV of 9 µg/L were based on one tablet per day for acetaminophen, while the higher LTD of 22.5 mg/kg-d (LTD_2_) and WSV of 50 µg/L were based on six tablets per day for acetaminophen. Both dosing regimens are therapeutically relevant and therefore were both included as comparison values.

## Supplementary Materials

The following are available online at http://www.mdpi.com/1660-4601/15/7/1308/s1, Table S1: Active Pharmaceutical Ingredient (API) Rapid Assessment Method Inputs, Decisions, Values, and Supporting Information.
